# Multidrug transporters: recent insights from cryo-electron microscopy-derived atomic structures and animal models

**DOI:** 10.12688/f1000research.21295.1

**Published:** 2020-01-13

**Authors:** Sabrina Lusvarghi, Robert W. Robey, Michael M. Gottesman, Suresh V. Ambudkar

**Affiliations:** 1 Laboratory of Cell Biology, Center for Cancer Research, National Cancer Institute, National Institutes of Health, 37 Convent Dr., Bethesda, MD, 20892, USA

**Keywords:** ABC transporter, P-glycoprotein, ABCG2, drug resistance, blood-brain barrier, ATP hydrolysis, cryo-EM, zebrafish

## Abstract

P-glycoprotein, ABCG2, and MRP1 are members of the ATP-binding cassette (ABC) transporter superfamily that utilize energy from ATP-binding and hydrolysis to efflux a broad range of chemically dissimilar substrates including anticancer drugs. As a consequence, they play an important role in the pharmacokinetics and bioavailability of many drugs; in particular, their role in multidrug resistance in cancer cells as well as at the blood–brain barrier has been the subject of studies for decades. However, the atomic structures of these transporters in the presence of substrates or modulators and at different stages of the ATP-hydrolysis cycle have only recently been resolved by using cryo-electron microscopy. In addition, new animal models have shed new light on our understanding of the role of these transporters at the blood–brain barrier. This new information should open doors for the design of novel chemotherapeutics and treatments to bypass recognition by ABC drug pumps to overcome clinical drug resistance. In this review, we discuss the most recent advances in our understanding of ligand interactions and mechanistic aspects of drug transport based on atomic structures of these transporters as well as the development of new *in vivo* models to study their role in clinical drug resistance in cancer.

## Introduction

Multidrug resistance (MDR)—resistance to multiple, structurally unrelated compounds—frequently arises during the treatment of cancer with chemotherapeutic agents. There are a number of ways by which cells can become resistant to multiple drugs, including drug inactivation, increased DNA repair mechanisms, activation of anti-apoptotic pathways, and increased drug efflux ^1^; the multifactorial nature of clinical cancer drug resistance has recently been reviewed ^2^. It is this latter mechanism that our review will explore in detail. Increased efflux of chemotherapy drugs usually occurs via increased expression of ATP-binding cassette (ABC) transporters, membrane transporters that use energy derived from ATP hydrolysis to efflux drugs from the cell against a concentration gradient. There are three main ABC transporters that contribute to MDR, and all were discovered via drug-resistant cell lines developed by incubating cancer cell lines with increasing concentrations of chemotherapy drugs.

The history of drug transporters began with studies by June Biedler, who described Chinese hamster cells selected in actinomycin D that were also resistant to daunomycin, vincristine, and vinblastine ^3^. Dano later demonstrated active transport of daunomycin from multidrug-resistant mouse Ehrlich ascites cells ^4^. The plasma membrane protein responsible for the observed MDR was termed “permeability glycoprotein” P-glycoprotein (P-gp) by Ling and colleagues ^5^, and the human gene that encoded P-gp, *MDR1* (later renamed *ABCB1*), was cloned in 1986 ^6^. The role of P-gp in MDR has been extensively characterized, and this transporter is known to transport a plethora of chemotherapy agents, including vincristine, vinblastine, doxorubicin, daunorubicin, bisantrene, mitoxantrone, and taxol as well as a number of kinase inhibitors ^7^.

The second transporter associated with MDR, the MDR-associated protein or MRP, was cloned in 1992 by Cole and colleagues from a doxorubicin-selected lung cancer cell line ^8^. Encoded by the *MRP* gene (later termed MRP1 and encoded by *ABCC1*), MRP1 was found to confer resistance to a more limited range of chemotherapy agents, including daunorubicin, vincristine, vinblastine, and etoposide ^9^. Subsequently, this transporter was shown to also transport glutathione-conjugated drugs and other compounds ^8^.

The third transporter associated with MDR, cloned by three separate laboratories nearly simultaneously, became known as breast cancer resistance protein (BCRP) ^10^, the ABC transporter expressed in the placenta (ABCP) ^11^, or the mitoxantrone resistance protein (MXR) ^12^. Overexpression of the gene that encodes for the ABCG2 transporter has been shown to confer resistance to a range of drugs, including mitoxantrone, topotecan, irinotecan, and a number of targeted kinase inhibitors ^13^. The half-transporter ABCG2 has a unique topology that includes an ATP site followed by a transmembrane domain (TMD) with six helices. Its functional unit is a homodimer ^14^.

Although discovered as mediators of drug resistance in multidrug-resistant cancer cells, these transporters serve important normal physiological transport functions in drug excretion from the body and in barrier functions such as at the blood–brain barrier (BBB). In this review, we summarize recent advances in the ABC transporter field with emphasis on recent insights into the atomic structure of the transporters, which may lead to the development of novel inhibitors of the transporters. Alternatively, these structures may be used to design chemotherapeutics that are not subject to transport. Additionally, we present recent findings using new animal models that may be used to evaluate the role of transporters at the BBB and to evaluate new treatments to bypass their role in drug resistance.

## Advances in the structure and conformational landscape of ABC drug transporters

ABC drug transporters use the energy from ATP binding and hydrolysis to pump cancer drugs out of cells before they reach their molecular targets ^15– 17^. Whereas P-gp exports hydrophobic substrates that are xenobiotics, MRP1 transports amphipathic organic acids (negatively charged, generally conjugated to glutathione, glucuronic acid, or sulfate) with large hydrophobic groups that are endobiotics as well as xenobiotics ^1^. ABCG2 preferentially transports xenobiotics and sulfated conjugates of steroids ^1^.

High-resolution structures of ABC drug transporters solved by cryo-EM in the last three years have provided invaluable information on the structural organization, ligand or modulator-binding, and transport mechanism of this family of proteins, as shown in Table 1. Such structures include inward-facing conformations of P-gp, MRP1, and ABCG2 with and without modulators and/or antibodies ^14, 18– 22^ as well as in the ATP-bound outward-facing conformation ^23– 25^.

**Table 1.** Atomic structures of various multidrug-resistance-associated mammalian ATP-binding cassette (ABC) transporters recently resolved by cryo-electron microscopy.

Protein	Ligand/nucleotide/antibody-FAB	Source	Resolution (Å)	PDB ID	Ref.
P-gp (ABCB1)	UIC2-Fab	Mouse/human-S559C/S1204C	4.14	6FN4	19
P-gp (ABCB1)	UIC2-Fab, Zosuquidar	Mouse/human S559C/S1204C	3.58	6FN1	19
P-gp (ABCB1)	UIC2-Fab, Taxol	Human	3.6	6QEX	20
P-gp (ABCB1)	UIC2-Fab, Zosuquidar	Mouse/human-E-Q	3.9	6QEE	20
P-gp (ABCB1)	ATP	Human-E556Q/E1201Q	3.4	6C0V	23
MRP1 (ABCC1)	Apo	Bovine	3.5	5UJ9	21
MRP1 (ABCC1)	LTC _4_	Bovine	3.3	5UJA	21
MRP1 (ABCC1)	ATP	Bovine-E1454Q	3.1	6BHU	24
ABCG2	5D3-Fab, Cholesterol	Human	3.8	5NJ3	14
ABCG2	5D3-Fab, E _1_S	Human-E211Q	3.58	6HCO	25
ABCG2	5D3-Fab, MZ29	Human	3.1	6ETI	22
ABCG2	MZ29	Human	3.56	6FFC	22
ABCG2	ATP	Human-E211Q	3.09	6HBU	25

E _1_S, estrone-3-sulfate; P-gp, P-glycoprotein.

### Overall structure

**Figure 1.**
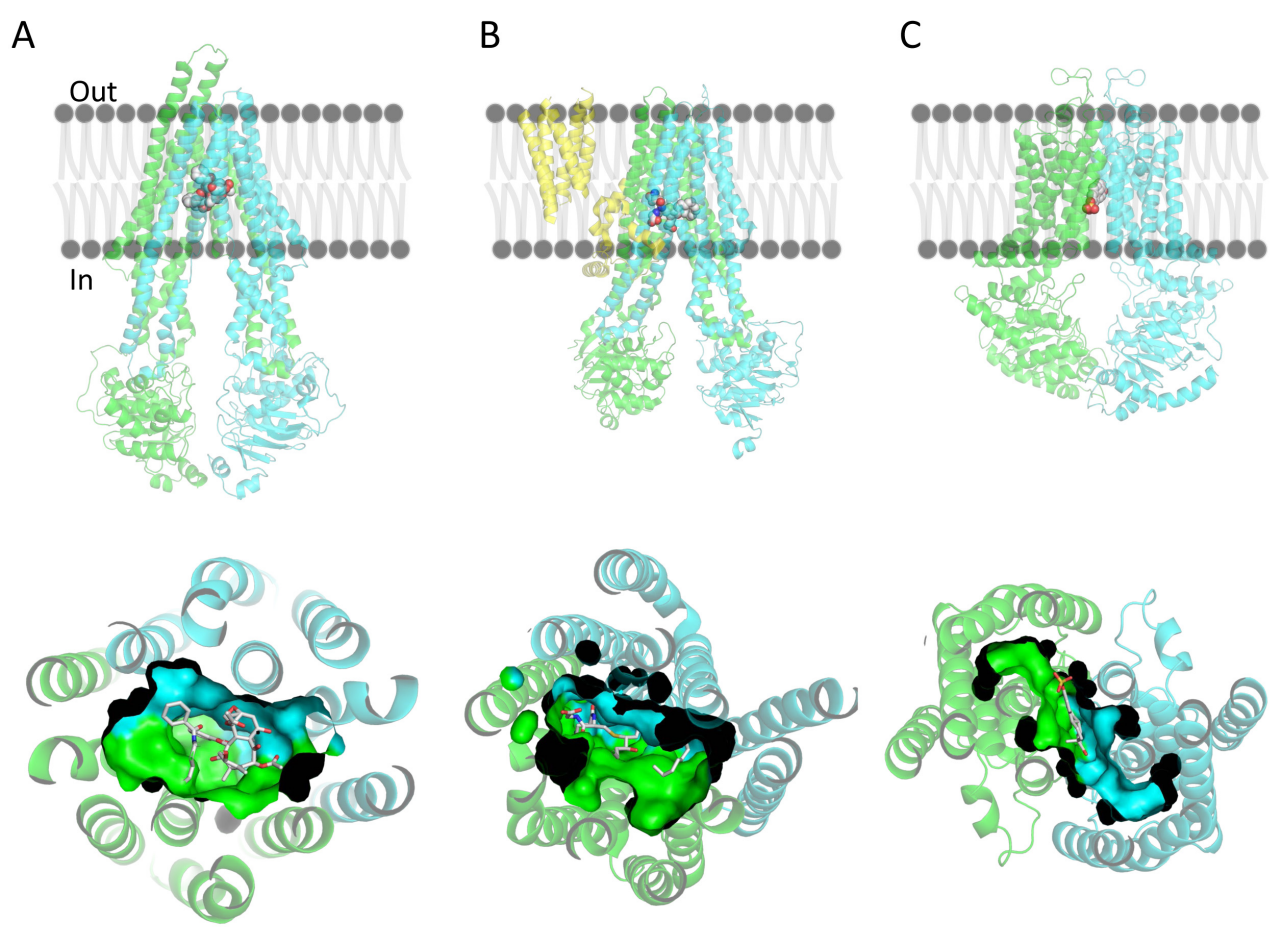
The overall structure of ( **A**) taxol-bound P-glycoprotein (P-gp), ( **B**) leukotriene C4-bound multidrug resistance protein 1 (MRP1), and ( **C**) estrone-3-sulfate-bound ABCG2. Cartoon representation of the different transporters on the top panels and cartoon and surface representation of the binding cavity as observed from the cytosolic region in the bottom panels. TMD0 of MRP1 is colored in yellow, the N-terminal half of P-gp and MRP1 (transmembrane domain [TMD] 1 and nucleotide-binding domain [NBD] 1) are colored in green, and the corresponding C-terminal halves (TMD2 and NBD2) are colored in cyan. Each monomer of the homodimer of ABCG2 is colored in green or cyan. Ligands bound in the transmembrane region are shown in ball and stick format (gray, carbon; red, oxygen; blue, nitrogen).

ABC transporters in general have a symmetric or pseudosymmetric structure, with two canonical TMDs connected by a flexible linker and two nucleotide-binding domains (NBDs) ^26^. The TMDs are involved in substrate recognition and translocation, whereas the NBDs bind and hydrolyze ATP. The structure can be a single polypeptide chain, as in the case of P-gp and MRP1 ( Figures 1A and 1B, respectively), or it could be a homodimer, as is the case for ABCG2 ( Figure 1C). Additional features further divide the ABC transporters into sub-families. For example, MRP1, which belongs to the ABCC family, has an additional TMD in the N-terminus (TMD0). Clearly, the TMD0 is not involved in drug–substrate recognition or translocation ( Figure 1B). P-gp consists of two TMDs and two functional NBDs. ABCG2, a homodimer, has two identical TMDs and NBDs, and the intracellular loops connecting the TMD to the NBD are shorter than in P-gp or MRP1, resulting in shorter distances between the NBDs as well as between the NBDs and the cell membrane ( Figure 1C).

The structure of mouse P-gp (mP-gp; 87% identical to human P-gp) has been solved by different groups using X-ray crystallography ^27– 30^. In general, the flexibility of the mP-gp molecule has yielded structures with lower resolution ^27– 29^, while shortening the linker region has increased the resolution by decreasing the mobility of the protein ^30^. Human P-gp seems to have a shorter distance between the NBDs when compared to its mouse counterpart, and only cryo-EM has yielded structural information ^20, 23^. In contrast, MRP1 and ABCG2 structures were unknown until their cryo-EM structures were solved, except for certain specific domains ^31, 32^.

**Figure 2.**
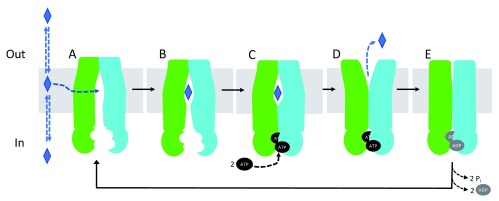
Proposed P-glycoprotein (P-gp) transport cycle. Substrates are presented as blue diamonds. ATP and ADP are presented in black and grey ovals. ( **A**) P-gp in the ligand-free, inward-open conformation. ( **B**) Ligand binds to the transmembrane region. ( **C**) ATP binds to the nucleotide-binding domains (NBDs) and favors their dimerization (outward-occluded conformation). ( **D**) The substrate is effluxed (outward-open conformation) and ATP is hydrolyzed to ADP and inorganic phosphate. Inorganic phosphate and ADP are released ( **E**), and the molecule is reset to the inward-open conformation.

The distance between the NBDs changes during the ATP-binding and hydrolysis cycle, resulting in a change in the conformation of the molecule from inward-facing to outward-facing. The ATP-dependent transport cycle of P-gp is summarized in Figure 2. Briefly, substrates bind in the transmembrane region of P-gp, directly from the membrane ( Figure 2A, 2B). ATP binds to the NBDs in the cytosol, favoring NBD dimerization. Conformational changes occur upon ATP binding that bring the NBDs closer together and prevent the release of substrates to the cytosol ( Figure 2C). Changes in the distance between the NBDs are translated to conformational changes in the TMDs that allow the translocation of molecules from the cytosol to outside the cell ( Figure 2D). ATP hydrolysis occurs at the interface of the NBDs, when they are in closest proximity to each other ( Figure 2E). After ATP hydrolysis, the molecule is reset to the inward-open conformation.

ABCG2 as well as P-gp structures have been solved in the presence of the 5D3- and UIC2-Fab antibodies, respectively. They both include extracellular loops that link the transmembrane helices (TMHs) together, known to be important in terms of blocking the back-influx of substrates as well as for antibody recognition ^14, 19, 21^. While two molecules of 5D3 bind symmetrically to ABCG2, a single UIC2 binds asymmetrically to P-gp. Binding of 5D3 prevents the formation of the outward-open conformation. The binding of the antibodies provides structural insights into the extracellular loops in the transporters, with flexibility constrained by the binding. Additionally, increasing the size of the transporters by way of the antibodies has facilitated the resolution of the structure by cryo-EM.

### Transmembrane domains

In the case of MRP1 and P-gp, TMD1 and TMD2 contain six TMHs each, arranged in two pseudosymmetric bundles. Bundle one contains the first three and the sixth TMHs of TMD1 and the fourth and fifth TMHs of TMD2, while bundle two contains the remaining TMHs. In the case of ABCG2, each bundle of six TMHs is composed of a monomer. A large transmembrane vestibule is located at the interface of the two bundles, opening to the cytoplasm and penetrating halfway into the lipid bilayer.

TMD0, present in MRP1, has been resolved at a lower resolution when compared to the other TMDs, indicating some flexibility. Interestingly, deletion of TMD0 does not affect transport or ATP hydrolysis ^33^. Although it may be important for interaction with other proteins, its functional partners have not yet been discovered ^21, 33, 34^. The TMD0 structure consists of five TMHs followed by an interfacial domain (lasso motif). Unlike TMD0, the lasso motif is highly conserved and essential for MRP1 folding and function, as it folds against TMH7, TMH15, and TMH16 at the membrane–cytosol interface. The cystic fibrosis transmembrane regulator (CFTR, ABCC7) has a lasso motif that resembles the motif in MRP1, indicating its common importance for folding and trafficking of some of the members of this sub-family ^35^.

### Substrate recognition

Substrates are believed to enter the drug-binding pocket of P-gp or ABCG2 directly from the membrane after partitioning into the inner leaflet of the lipid bilayer or directly from the cytoplasm ^36^. The helices in the TMD of P-gp can undergo rotational and translational movements to accommodate substrate entry ^30^. In contrast, substrates for MRP1 can enter only through the cytoplasm ^21^.

The changes in the TMHs during the ATP hydrolysis cycle—rotation, translocation, and unwinding—are also reflected in the translocation pathway, supporting the idea of an induced fit model in which the substrate creates its own binding site by interacting with different residues from different TMHs within the translocation pathway, utilizing a number of different interactions including hydrophobic, aromatic, van der Waals, and hydrogen bonds ^30^. The presence of ligands inside the binding site provides invaluable information (simplified in Figure 2B). For example, MRP1 substrate binding has revealed local as well as global conformation changes ^21^. The two transmembrane bundles rotate, moving the NBDs 12 Å closer in the presence of LTC _4_. This conformational change also explains the increased ATP hydrolysis, given that the NBDs are closer and better aligned. LTC _4_ interacts through hydrogen bonds and van der Waals interactions with both transmembrane bundles. The binding site has a positively charged region that coordinates the GSH moiety (P-pocket) and a large hydrophobic area that interacts with the lipid tail (H-pocket) ^24^. The P-pocket is formed by residues of both transmembrane bundles, whereas the H-pocket is formed by residues from transmembrane bundle 2. Most of LTC _4_ is buried inside the cavity, except for a carboxylate that points towards the cytoplasm. The H-pocket is larger than the LTC _4_ lipid tail, allowing the binding site to accommodate a variety of substrates. In addition, there are a few polar side chains that allow interaction with polar groups from otherwise hydrophobic substrates. Many residues undergo local rearrangement to accommodate the LTC _4_ binding. The structure of MRP1 in the presence of ATP in the closed conformation reveals that LTC _4_ is effluxed out of the protein prior to ATP hydrolysis ^24^. It also reveals movement of amino acids in the transmembrane region that forces the substrate out of the binding site by weakening the binding interaction and increasing steric hindrance ^23^.

P-gp transports hydrophobic or amphipathic compounds and some weakly positively charged substrates, in contrast to MRP1, which transports mostly organic acids ^1^. The translocation pathway in P-gp is mostly hydrophobic with some acidic patches, whereas the one in MRP1 is mostly basic. The structure of intermediate conformations of human P-gp bound to the substrate Taxol and the atomic structure of chimeric mouse–human P-gp bound to the inhibitor zosuquidar has helped in understanding the adaptability, plasticity, and polyspecificity of the binding site of this transporter, supporting the model of an induced fit mechanism for ligand binding ^19, 20^. Particularly, two molecules of zosuquidar are found in the binding pocket of mouse P-gp, occupying most, but not all, of the empty space ^19^. Similar to MRP1, residues from eight out of 12 TMHs interact with the ligands ^20^. Interestingly, two TMHs, TMH4 and TMH10, seem to play an important role in ligand binding to P-gp by alternating from a straight to bent to straight helix as the ligand binds and is translocated through the transmembrane region ^20^. The helix breakers in these helices seem to be essential to block the release of the substrate to the cytosol, generating a closed gate in the bound conformation as well as a continuum helix upon ATP binding, likely favoring the unidirectional movement of the substrate. The movement of these helices as well as TMH6 and TMH12 also blocks the lateral gate (portal) through which ligands are presumed to enter from the membrane. Structures of the ATP hydrolysis-deficient E-Q mutant of chimeric P-gp bound to zosuquidar in the absence or presence of ATP render a molecule with the two NBDs separated, suggesting that zosuquidar inhibits P-gp function by preventing the dimerization of the NBDs ^19, 20^. Double electron-electron resonance (DEER) spectroscopy has been used to study the energy landscape of the interaction between stimulators of ATP hydrolysis and inhibitors, and it further supports the prevention of dimerization of NBDs by binding of inhibitors in the transmembrane region ^37^.

In contrast to MRP1 and P-gp, in which the binding cavity is wide and flexible, the cavity in ABCG2 is delimited by TMH1, TMH2, and TMH5 from both monomers and seems to be narrower, which likely sterically restricts substrates that can bind in the binding pocket ( Figure 1C, bottom). The cavity opens to the cytoplasm and the inner leaflet of the lipid bilayer, and it can accommodate flat, polycyclic, and hydrophobic substrates ^14^. In addition, there is a second cavity that is accessible only upon conformational changes in the first one. It has less hydrophobic surface area, which might lead to lower substrate affinity, favoring the release of the substrate on the extracellular side during transport. Cholesterol ^14^, the substrate estrone-3-sulfate (E _1_S) ^25^, as well as two different inhibitors, MB136 (a tariquidar derivative) and MZ29 (a Ko143 derivative) ^22^, have all been shown to bind to the first cavity. The structure of ABCG2 bound to cholesterol and MZ29 revealed two molecules in the binding cavity, whereas for E _1_S or MB136 only a single molecule was bound. Interestingly, the lower density found for the latter compounds suggests that these molecules may have more than one way of interacting with the binding site. Specific mutation of the amino acids found to interact with E _1_S provides insights into the role of each residue for the binding of the substrate ^25^.

Although the structures of ABCG2 and P-gp with substrates and inhibitors bound in the transmembrane region have been solved, it is still not completely clear what exactly makes a molecule become an inhibitor or a stimulator of ATPase activity. Clearly, higher affinity, as a result of more local interactions and better coverage of the binding cavity, seems to be the driving force that distinguishes between a stimulator and an inhibitor by locking the transporters in a conformation in which the two NBDs can no longer dimerize ^20, 22, 25^.

### NBD–TMD interface and NBD1–TMD2 linker

In general, the interfaces of the transporters do not seem to change significantly upon ATP binding; they move as rigid motifs accompanying NBD movement. In the case of ABCG2, the linker, which is an extension of TMH1, is much shorter and highly charged, yet the structure of this region remains unknown, suggesting high flexibility.

When the structure is a single polypeptide chain, such as in the case of P-gp and MRP1, the NBD1 and the TMD2 are connected by a flexible linker. Even though this linker is essential for proper function, it generally appears unstructured in most of the ABC transporter structures resolved to date ^19– 21, 23, 24^.

### Nucleotide-binding domains

In all three transporters, when ATP is bound, the NBDs dimerize and the TMDs open to the extracellular region, referred to as the inward-closed or outward-facing conformation. In the inward-open conformation, the two NBDs are separated from each other; however, during the transport cycle, they dimerize in a “head-to-tail” configuration to form two combined ATPase sites, with the Walker A/B motifs forming one NBD and the signature motif of the other. Interestingly, ABCG2 does not have an A-loop ^22^. The adenine is stacked by an aromatic residue, as is found in other transporters. Rather, van der Waals interactions and salt bridges compensate for the binding ^25^. ATP hydrolysis is catalyzed via a general base mechanism in which a glutamate residue polarizes the hydrolytic water for inline attack of the ATP-γ-phosphate. Mutations to the highly conserved Walker B catalytic glutamate residue render P-gp, MRP1, and ABCG2 capable of binding ATP but not hydrolyzing it. However, the binding of transport substrate is not altered ^38^. These E-Q mutants have been informative in solving the ATP-bound closed conformations of several ABC transporters ^23– 25, 39^.

Cryo-EM studies performed on purified P-gp in detergent micelles complexed with and without UIC2-Fab at different stages of the ATP hydrolysis cycle have provided key information on the movement of the NBDs during this process. Structures have been reported for the unbound state (apo), ATP-bound before hydrolysis, ADP-V _i_-bound post hydrolysis, and ADP-bound after P _i_ release ^18, 19, 23^. The apo form was found to be present in the inward-facing conformation as well as in an outward-facing conformation. The ATP-bound pre-hydrolysis sample yielded a mixture of closed and open conformations in a different proportion compared to that observed in the apo structure ^18^. ATP-bound after hydrolysis and trapped in the transition state with V _i_ replacing the outgoing P _i_ yielded mostly a closed structure with the two NBDs in the dimerized conformation ^18^. Lastly, the post-hydrolysis and after release of the P _i_ states yielded structures in mostly the open conformation, suggesting that the presence of the ADP prevents the closing of the structure ^18^. The structure of the ATP-bound outward-facing conformation suggests that the substrate is effluxed from the drug-binding cavity before ATP is hydrolyzed and that the energy from the ATP hydrolysis is essential for restoring the inward-facing conformation ^23^. This proposed mechanism is based on the atomic structure of the ATP-bound E-Q mutant transporter ^23^. This needs further validation by obtaining the high-resolution structure of the ATP-bound wild-type transporter in the presence and absence of a transport substrate.

In P-gp, both NBDs are highly homologous and capable of hydrolyzing ATP; however, they depend on each other for catalysis. A recent crystal structure shows preferential binding of ATP NBD1 in the shorter linker mP-gp, supporting the hypothesis of asymmetric binding ^30^. Only one ATP hydrolysis can occur at a time; inactivation of one of the sites inactivates ATP hydrolysis. How the two ATP-binding sites alternate remains unknown. DEER spectroscopy was used to study spin-labeled mutants in a variety of conditions ^40^. Distance distributions between each different pair of labels agree with a model that fluctuates between an inward-facing and an outward-facing conformation, with extensive conformational changes during the transition state. In this state, the distribution between intracellular labels was homogeneous, whereas the distribution in the extracellular region was more variable, highlighting a reconfiguration of the TMDs. Labels on the NBDs showed structural asymmetry and supported the alternating site hydrolysis model, in which two-stage hydrolysis is required to translocate drugs outside the cell ^40^. In contrast, MRP1 has NBD1 with significantly lower ATPase activity compared to NBD2 ^21, 35^. Cryo-EM structures of MRP1 in the apo, ligand-bound, and ATP-bound conformations show three different distances between the NBDs. Transmembrane bundle 1 and NBD1 as well as transmembrane bundle 2 and NBD2 move, bringing the NBDs closer together. A substrate can bind to the protein only before ATP binds, and binding of the substrate brings the NBDs closer together, resulting in enhanced ATPase activity. On the other hand, ABCG2 has shorter intracellular loops, resulting in shorter distances between the NBDs, which remain in contact despite the absence of nucleotide.

Based on the proposed mechanism described in Figure 2D, the substrate is released prior to ATP hydrolysis, suggesting that ATP hydrolysis is not needed for the transport function of P-gp but rather to reset the molecule to the inward-open conformation ( Figure 2E). The residues that are found to interact with the ligands collapse, leaving no room for the substrate to be bound. In order to be released, the substrate has to be pushed towards the extracellular region, to a region of the protein where the affinity of the substrate is decreased. Additional high-resolution structures of wild-type transporters in different conformations including ATP- and ligand-bound will help to understand the role of ATP hydrolysis in the transport cycle.

The binding of ATP to the NBDs is accompanied by changes in the transmembrane region that result in a completely blocked binding site and translocation pathway. In contrast to the bacterial homodimer multidrug transporter (Sav1886), these transporters do not show a much wider outward-open conformation upon ATP binding ^41^. The structure instead corresponds to a narrow translocation pathway for MRP1, P-gp, and ABCG2 that is protected from the lipid bilayer ^23– 25, 39^.

### Membrane environment

The importance of a lipidic environment for transporter function has been previously shown ^30, 42, 43^. Cryo-EM structures of P-gp and ABCG2 have identified ordered cholesterol and phospholipid molecules directly interacting with the transmembrane region ^20, 22^, suggesting that the lipids in the membrane could modulate the conformational changes associated with binding of substrates and inhibitors. In addition, it has been demonstrated that P-gp inhibitors no longer inhibit ATP hydrolysis when the protein is in detergent micelles ^44^, whereas the inhibition is recovered when the protein is reconstituted in nanodiscs or proteoliposomes. Also, it has been shown for the MsbA transporter that the distance between the NBDs is larger when the protein is in detergent micelles ^45^. These findings highlight the importance of using a membrane environment for structural studies ^42, 43^ and show that purified protein in a detergent micelle environment is not suitable for studying the interaction of substrates and high-affinity modulators with P-gp. As all structural studies with MRP1 have been done in detergent micelles ^21, 24^, it will be of interest to see if the same observations hold true for MRP1 reconstituted in nanodiscs.

## Animal models to study transporters at the blood–brain barrier

While atomic structures of transporters and molecular modeling can help to identify novel inhibitors or potentially develop compounds that are not subject to ABC transporter-mediated efflux, *in vivo* models will eventually be needed to test the compounds, particularly when evaluating the role of transporters at the BBB.

**Figure 3.**
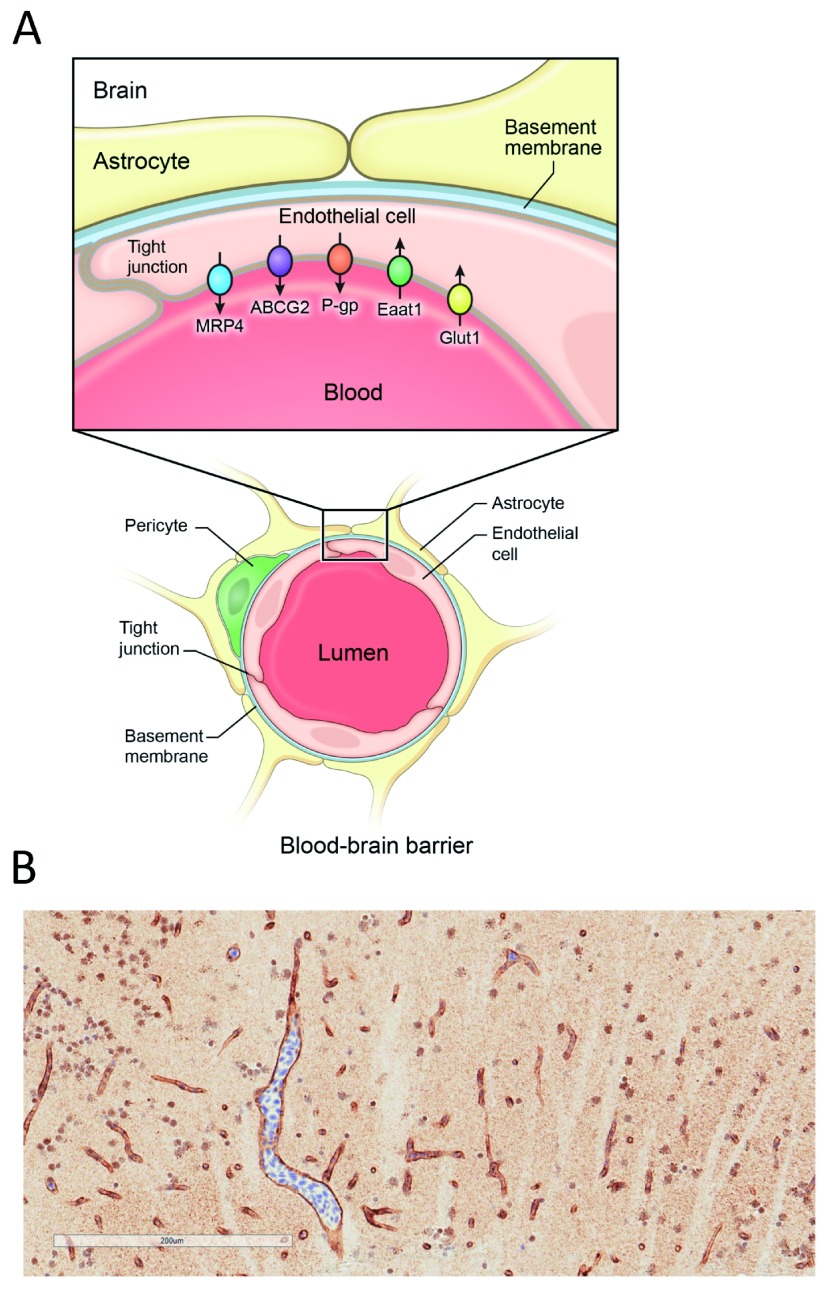
**A**. Schematic representation of the blood–brain barrier. The blood–brain barrier is formed primarily by brain endothelial cells in capillaries and is regulated by surrounding pericytes and astrocytes at the basolateral side of the endothelial cells. The endothelial cells form tight junctions, mediated by connexin, occludin, and claudin family proteins. At the apical cell surface, ABC transporters such as P-gp (P-glycoprotein, ABCB1), ABCG2 (also breast cancer resistance protein), and MRP4 (multidrug-resistance protein 4, ABCC4) transport small molecules back into the lumen. Ingress of nutrients from the blood supply is mediated by facilitative solute carrier SLC transporters, such as glutamate (excitatory amino acid transporter 1, Eaat1, SLC1A3) and D-glucose (glucose uptake transporter 1, Glut1, SLC2A1). Lining the apical surface and projecting into the lumen is the glycocalyx (not shown), composed of glycoprotein and polysaccharide. This panel was reprinted by permission from *Springer Nature* ^46^: *ABC Transporters - 40 Years On*, Basseville *et al.*, The ABCG2 multidrug transporter, 2016. [ https://link.springer.com/chapter/10.1007/978-3-319-23476-2_9]. **B**. Zebrafish homologs of human P-glycoprotein (P-gp) are expressed at the blood–brain barrier. Expression of zebrafish homologs of P-gp were determined by immunohistochemistry staining with the C219 antibody on formalin-fixed, paraffin-embedded zebrafish. Staining is found in the vasculature of the zebrafish brain.

As shown in Figure 3A, the BBB is made up of endothelial cells that provide a physical barrier in the form of tight junctions which limit the diffusion of compounds from the bloodstream into the brain ^7, 46^. ABC transporters provide a second line of protection by transporting compounds back into the bloodstream against a concentration gradient, thus protecting the central nervous system ^47^. P-gp and ABCG2 are two of the transporters that are most highly expressed at the BBB. Owing to the ever-increasing number of compounds that these two transporters can efflux, they are a significant impediment to chemotherapeutic treatment of the brain ^47^.

While some *in vitro* models have been developed to determine whether transporters at the BBB can transport drug candidates, they do not accurately model the complexity of the BBB ^48^. As such, mouse models have been instrumental in determining the role of transporters in preventing access of chemotherapeutics to the brain ^48^. However, the zebrafish has recently been proposed as an alternative model ^49^.

The important contribution of P-gp at the BBB, because of high expression in brain endothelial cells ^50^, was first revealed with the development of mice that were deficient in *Abcb1a* and *Abcb1b*, the murine homologs of human *ABCB1*. Schinkel and colleagues made the serendipitous discovery that mice that lacked Abcb1a and Abcb1b expression were exquisitely sensitive to the antiparasitic drug ivermectin ^51^. Subsequently, P-gp was found to prevent brain penetration of several drugs including vinblastine, dexamethasone, digoxin, and cyclosporine A ^51, 52^. Shortly after its discovery, ABCG2 was also found to be highly expressed in brain endothelial cells ^53^. When mice lacking *Abcb1a*, *Abcb1b*, and *Abcg2* were generated, a compensatory and perhaps synergistic role for the transporters at the BBB emerged, particularly for kinase inhibitors that are used as targeted cancer therapies ^7^. In one recent example, brain concentration of ponatinib, an inhibitor of the BCR–ABL1 fusion kinase, was found to be 2.2-fold higher in Abcg2-deficient mice than in wild-type mice, 1.9-fold higher in Abcb1a/1b-deficient mice, and 25.5 fold higher in mice lacking all three transporters ^54^. Similarly, for afatinib, a dual EGFR/HER-2 inhibitor, brain concentration of the drug was 4.6-fold higher in Abcg2-deficient mice compared to wild-type, 3.2-fold higher in Abcb1a/1b-deficient mice, and 1,208-fold higher in mice deficient for all three transporters ^55^. The fact that brain endothelial cells form tight junctions severely limits passive diffusion across the BBB, leading to the apparent synergistic role of these transporters at the BBB ^56, 57^. It is clear from the studies with knockout mice that it will be necessary to inhibit both P-gp and ABCG2 transporters in order for substrate compounds to enter the brain, as co-administration of kinase inhibitors with the dual P-gp/ABCG2 inhibitor elacridar can mimic the increased brain penetration observed when both P-gp and ABCG2 are knocked out ^58^.

In addition to the knockout mice, other models have been used to study inhibition of transporters at the BBB ^7^. We found that D-luciferin, the substrate of firefly luciferase, is specifically transported by ABCG2 ^59^. Capitalizing on this fact to investigate the role of ABCG2 at the BBB, we used a transgenic mouse model that expresses firefly luciferase under the control of the glial fibrillary acidic protein (GFAP) promoter, leading to expression of luciferase in the astrocytes ^60^. When these mice are administered D-luciferin, the action of ABCG2 prevents luciferin from crossing the BBB and interacting with the luciferase expressed in the astrocytes ^60^. When the luciferin is co-administered with an inhibitor of ABCG2, such as Ko143, the luciferin is able to cross the BBB and react with luciferase, thus generating a light signal in the mouse brain ^60^. This model could be used to test potential inhibitors of ABCG2 for their ability to increase brain penetration of chemotherapy.

Another animal model that has gained interest as an alternative to the mouse is the zebrafish ^61^. Zebrafish have a BBB comprising endothelial cells that form tight junctions that express zona occludens-1 and claudin-5 ^62^. Although zebrafish have no direct homolog of human *ABCB1*, they have two genes that are similar, *abcb4* and *abcb5* ^63, 64^. The anti-P-gp monoclonal antibody C219 recognizes both zebrafish Abcb4 and Abcb5, and this antibody has been used to localize zebrafish homologs of P-gp to the zebrafish BBB ^62^; however, it is not known if one or both zebrafish homologs are expressed at the BBB. We also observed staining with the C219 antibody in the vasculature of the zebrafish brain by immunohistochemistry ( Figure 3B). In terms of substrate specificity, Abcb4 has been shown to transport some known fluorescent P-gp substrates ^64^, and Abcb5 was recently found to be responsible for the transport of fluorescent P-gp substrates in zebrafish embryo ionocytes ^65^. Apart from these few reports, the substrate specificities of the zebrafish transporters homologous to P-gp have not been thoroughly explored.

Much less work has been done with zebrafish homologs of ABCG2. Zebrafish have four genes that are homologous to *ABCG2*— *abcg2a*, *abcg2b*, *abcg2c*, and *abcg2d* ^63^. Zebrafish Abcg2a has been shown to transport Hoechst 33342 ^66, 67^, but little is known about other substrates of Abcg2a and no information has been reported on the other homologs. Additionally, none have been localized to the zebrafish BBB, most likely because of a lack of antibodies specific to the zebrafish proteins. The power of using zebrafish as a model to study transporter inhibitors or to examine the ability of therapies to cross the BBB is an exciting possibility.

## Conclusion

The important physiological role of ABC drug transporters, especially those with very broad substrate specificity such as ABCB1, ABCC1, and ABCG2, has become clear over the past few decades. Major advances in structure–function studies in recent years have begun to reveal common and unique features of the mechanism of polyspecificity and transport cycle of these transporters. Simultaneously, genetic and physiological studies have emphasized the important excretory and barrier functions of these transporters. In particular, their role in contributing to the BBB is beginning to be revealed through animal models such as the mouse and zebrafish.
